# Study of selected mechanisms of oat tolerance to cadmium and powdery mildew

**DOI:** 10.1007/s11356-025-36951-x

**Published:** 2025-10-15

**Authors:** Veronika Kubová, Vladimír Langraf, Libuša Lengyelová, Beáta Piršelová

**Affiliations:** 1 Department of Botany and Genetics, Faculty of Natural Sciences and Informatics, Constantine the Philosopher University in Nitra, 949 01 Nitra, Slovakia; 2 Department of Zoology and Anthropology, Faculty of Natural Sciences and Informatics, Constantine the Philosopher University in Nitra, 949 01 Nitra, Slovakia

**Keywords:** *Avena sativa*, *Blumeria graminis*, Co-tolerance, Defence, Metal

## Abstract

This study evaluates the tolerance mechanisms of different varieties of oats (*Avena sativa* L., vars. Aragon, Bay Yan 2, Ivory, Racoon, and Vaclav) to cadmium (50 mg·kg⁻^1^ soil applied on day 22, Cd treatment), the pathogen (*Blumeria graminis* f. sp. *avenae*, applied on day 35, P treatment), and their simultaneous effect (P + Cd treatment). Cd accumulation was generally higher in the infected plants (P + Cd) and depended on the genotype. The tested oat vars. were more sensitive to P than to Cd, which was reflected in a significant reduction (> 40%) in photosynthetic pigment content, an increase in polyphenol content, higher lipid peroxidation in membranes, and increased activity of total β-1,3-glucanase and catalase in infected leaves. A significant increase in calcium content was observed in the leaves of the P + Cd treatment. The reduced glutathione content in the Cd and P + Cd treatments (compared to the P treatment) in most of the tested vars. suggests different roles for glutathione in defence against Cd (metal sequestration) and P (antioxidant role). The reduction in lipid peroxidation, polyphenol content, and total β-1,3-glucanase activity in plants of the P + Cd treatment suggests a potential priming effect of cadmium on defence mechanisms. The obtained results have the potential to predict oat behaviour not only under cadmium stress or powdery mildew infection, but also under combined stress conditions.

**Supplementary Information:**

The online version contains supplementary material available at 10.1007/s11356-025-36951-x.

## Introduction

Plants are exposed to various types of abiotic and biotic stress throughout their life, and in the era of global warming, the mobility of pollutants in the environment is increasing. Therefore, it is extremely important to understand the mechanisms that underlie plant resistance or tolerance to various undesirable environmental factors. Plants are rarely exposed to just one type of stress; they usually have to cope with multiple stressors simultaneously. In a competitive growing environment, the ability of plants to survive depends on their capacity to recognize, integrate, and respond to both biotic and abiotic environmental variables through the constant adjustment of their physiology and metabolism. This ability is supported by cross-tolerance (Pastori and Foyer 2002; Mittler et al. 2006; Llorens et al. 2020; Nawaz et al. 2023), which is achieved through the synergistic co-activation of the plant’s innate immune system that involves a network of non-specific stress response pathways (Atkinson and Urwin 2012; Bostock et al. 2014). The plant response to the simultaneous action of multiple stressors can result from mutual antagonistic, synergistic, or neutral interactions (Poschenrieder et al. 2006; Malinovsky et al. 2014). Although plant resistance to metals has been widely studied, very few studies focus on assessing this stress under conditions of concurrent pathogen infection. Existing studies suggest plants’ common response mechanisms to defend against these stressors. Several studies have pointed out the protective effect of metals in conditions where plants are threatened by pathogens. Many plant species, such as hyperaccumulators, absorb high concentrations of metals from the soil as a defence mechanism against herbivores and pathogens (Poschenrieder et al. 2006; Llugan et al. 2019). The hypothesis that hyperaccumulation provides resistance to biotic stress was originally formulated based on observations that less insect activity occurs on nickel (Ni) hyperaccumulators. Further research has shown that high levels of nickel (Ni), zinc (Zn), cadmium (Cd), or selenium (Se) can offer effective protection against fungi or viruses. Hyperaccumulation can therefore facilitate cross-resistance to microbial pathogens. Several studies have indicated that defence in this case is not only mediated by the metal itself, but also by the metabolites synthesized in response to the metal in plants (Morkunas et al. 2018). In this context, defence can be ensured by polyphenols, alkaloids, oxylipins, etc. (Yao et al. 2012). Therapy with certain (low) metal doses is a mechanism known as priming, in which defence mechanisms effective against other stressors are induced (Morkunas et al. 2018). This mechanism has been observed during the pathogenesis of various plants exposed to metal stress (Mohapatra and Mittra 2017; Morkunas et al. 2018; Xu et al. 2023). It has been shown, for example, that the overexpression of the calcium-binding protein 1 (CABPR1) from pepper or fungal chitinase type PR3 can provide resistance to a broad range of fungal and bacterial pathogens, as well as to some abiotic stressors, such as Cd (Sarowar et al. 2005; Balasubramanian et al. 2012). The result of the metal-pathogen interaction is strongly determined by metal type, dose, and the plant’s tolerance to these stressors (Rivero et al. 2022; Nawaz et al. 2023). Although the specificity of resistance mechanisms to metal ion toxicity and biotic stress is well characterized, the significance of cross-communication in signalling pathways remains a debated topic. Responses to excess metal ions in sensitive plants can resemble defence reactions triggered by an elicitor (pathogen) (Vollenweider and Gűnthardt-Goerg 2005). Biotic signal transduction pathways offer several points of interaction with metal ion stress signalling. Stress-activated cytoplasmic calcium (Ca) levels increase, stimulating the production of reactive oxygen species (ROS), nitric oxide (NO), salicylates, thioredoxins, and mitogen-activated protein kinases (MAPKs), which represent key cross-points of interaction between pathogen-induced responses and more or less specific toxic effects of various metals (Glazebrook 2005; Amari and Niehl 2020). The interaction of certain signalling molecules during the action of metals and pathogens, and the ability to maintain high levels of reduced glutathione, antioxidant enzymes, and secondary metabolites, seems a key factor for co-tolerance (Romero-Puertas et al. 2004; Walters et al. 2005). However, it appears that signalling pathways are highly stress-specific (Glombitza et al. 2004). The specificity of the receptor, subcellular sites of ROS production, the specificity and regulation of MAPK activities (Pedley and Martin 2005), and differences in activated genes and their products are responsible for the specificity of responses.

Regarding the physiological mechanisms involved in adaptation to stress, it is well known that heavy metal stress increases levels of phytohormones (especially abscisic acid, salicylic acid, and jasmonic acid), which are significant in the induction of (for example) pathogenesis-related (PR) proteins and other biomolecules important in pathogen defence. Through PR-proteins, plants degrade microbial and pathogen cell components. Such degraded structures are defined as elicitors and represent conserved molecules specific to microbes, known as microbe- or pathogen-associated molecular patterns (MAMP or PAMP). They are recognized by pattern recognition receptors in the innate immune systems of plants, which lead to the activation of hypersensitive responses (HR) (Villada et al. 2009). As a result of activating various signalling pathways (e.g. via metals or pathogens), there is also cell wall strengthening through the synthesis of callose, hydroxyproline, glycoproteins, and phenolic compounds (including lignin and suberin) (Deepak et al. 2010).

The goal of this study was to (i) assess the variability in tolerance of selected oat varieties to a chosen dose of Cd and powdery mildew (*Blumeria graminis* f. sp. *avenae*), (ii) evaluate the mechanisms involved in defence against individual stressors and their combination, and (iii) assess the potential priming effect of Cd in the context of defence against powdery mildew.

## Material and methods

**Fig. 1:**
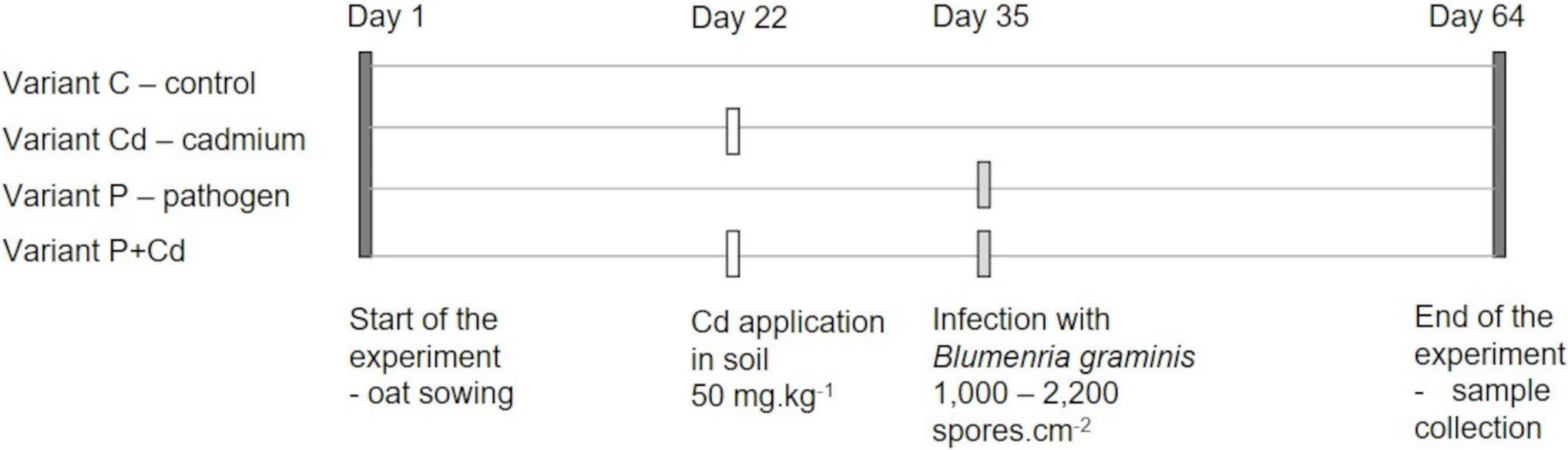
Design of the experiment

The experiment was designed as a pot trial conducted under laboratory conditions, following the methodology described by Piršelová et al. (2024). Seeds of five oat varieties (*Avena sativa* L., vars. Aragon, Bay Yan 2, Ivory, Racoon, and Vaclav) were sown into plastic pots (25 cm in diameter) filled with 1250 g of Klasmann TS 2 substrate. Plants were cultivated in a Fitotron II growth chamber under controlled environmental conditions: temperature range of 15–24 °C, relative humidity of 62–82%, a 16/8 h light/dark photoperiod, and a Light intensity of 20,000 lx, with ten plants per pot. After 22 days of growth (at the third leaf stage), Cd was applied to selected variants at a concentration of 50 mg Cd.kg⁻^1^ of substrate (as CdCl₂). On day 35, plants in the pathogen treatment variants were inoculated with the fungal pathogen *Blumeria graminis* f. sp. *avenae* using a settling tower, which delivered 1000–2500 spores.cm⁻^2^ to the leaf surface. In total, four experimental variants were established: (1) control (C), without Cd or pathogen; (2) cadmium (Cd), Cd applied on day 22; (3) pathogen (P), pathogen inoculated on day 35; and (4) cadmium + pathogen (Cd + P), Cd applied on day 22 and pathogen inoculated on day 35 (Fig. 1). All pathogen-infected plants were kept physically separated to prevent cross-contamination. On day 64 from the start of cultivation, leaf samples were collected for individual analyses.

### Measurement of photosynthetic pigment contents in leaves

The content of assimilation pigments was determined in acetone extracts according to Lichtenthaler and Wellburn (1983). The measured parameters were as follows: chlorophylls (Chl*a*, Chl*b*) and carotenoids (Car). The concentration of photosynthetic pigments was determined from absorbance peaks at 470, 663, and 646 nm, measured by a spectrophotometer (UV-2601). Acetone was used as a blank. Pigment concentrations (c, mg.L^−1^) were calculated using the following formulas :

$$\mathrm c\;\mathrm{Chl}a\:=\:12.{21}_{A663}\:-\:2.{04}_{A646}$$

$$\mathrm c\;\mathrm{Chl}b\:=\:20.{13}_{A646}\:-\:4.{19}_{A663}$$

$$\mathrm c\;\mathrm{Chl}\;a\:+\:b\:=\:7.{05}_{A663}\:+\:18.{71}_{A646}$$

$$\mathrm c\;\mathrm{Car}\:=\:({1000}_{A470}\:-\:3.27\mathrm c\;\mathrm{Chl}a\:-\:104\mathrm c\;\mathrm{Chl}b)/229$$

The concentration of each pigment was converted to mg.g^−1^ fresh weight (FW).

### Measurement of cadmium and calcium in plant tissue and soil

Plant material and soil sample (0.5 g each) were digested in a mixture of 5 mL water, 5 mL HNO_3_ (Merck, Darmstadt, Germany), and 1.5 mL H_2_O_2_ (30%) using a Mars Xpress microwave oven (CEM Corporation, Matthews, USA). Cd and Ca were detected by inductively coupled plasma optical emission spectroscopy (ICP-OES 725, Varian 725 ES ICP, Melbourne, Australia) according to Kováčik et al. (2018).

### Estimation of lipid peroxidation of cell membranes

Lipid peroxidation of leaf cell membranes was determined according to Heath and Packer (1968), based on the amount of malondialdehyde (MDA) in the samples. Fifty mg of plant material was homogenized in 1 mL of 5% trichloroacetic acid (TCA). The homogenate was centrifuged at 10,000 × g for 5 min. To each 400 µL aliquot of the supernatant, 1.5 mL of 20% TCA containing 0.5% thiobarbituric acid (TBA) was added. The mixture was heated at 90 °C for 60 min and then rapidly cooled in an ice bath. The resulting mixture was centrifuged again at 10,000 × g for 5 min, and the absorbance of the supernatant (1 mL) was measured at 532 nm. Measurements were corrected for non-specific turbidity by subtracting absorbance at 600 nm. The MDA concentration was determined using a calibration curve for MDA standard (Thermo Fisher Scientific).

### Measurement of catalase activity

Catalase (CAT) activity was determined using a CAT activity assay kit (Invitrogen), following the manufacturer’s instructions. The tissues (200 mg) were homogenized in 1 mL of diluted buffer-1X Assay Buffer and centrifuged at 10,000 × g for 15 min at 4 °C. Twenty-five microlitres of each sample or catalase standard was added to the wells of a 96-well microplate. The reaction was initiated by adding the substrate containing hydrogen peroxide (H₂O₂). Samples were incubated at room temperature for 30 min, during which catalase enzymatically decomposed H₂O₂ into water and oxygen. After incubation, a detection reagent mixture containing a chromogenic reagent and horseradish peroxidase (HRP) was added to each well. This mixture reacted with the residual (unconverted) H₂O₂, producing a pink-colored product. Following an additional 15-min incubation, absorbance was measured at 560 nm using a Multiskan Sky microplate spectrophotometer (Thermo Fisher Scientific). Reaction buffer without catalase was used as a negative control. Catalase activity was quantified using a calibration curve prepared from a standard bovine catalase solution (100 U·mL⁻^1^).

### Estimation of glutathione content in leaves

The total glutathione (GSH, γ-glutamyl-cysteinyl-glycine), reduced (2GSH), and oxidized glutathione (GSSG) content was determined spectrophotometrically using a GSH/GSSG kit (Invitrogen) with an enzymatic method utilizing Ellman’s Reagent (DTNB: 5,5′-dithiobis-(2-nitrobenzoic acid)) and glutathione reductase (GR). DTNB reacts with reduced glutathione to form a Yellow product. The rate of change in optical density, measured at 412 nm, is directly proportional to glutathione concentration in the sample. A specific kit protocol was used to determine GSSG, whereby all GSH was removed in the first stage using 1-methyl-2-vinylpyridinium triflate.

### Measurement of β−1,3-glucanase activity

Crude protein extracts were isolated from plant leaf tissue samples (200 mg) according to the method of Hurkman and Tanaka (1986). Protein concentration was determined according to Bradford (1976). Obtained extracts were aliquoted and stored for further analyses in a deep freezer at − 80 °C. The activity of beta-1,3-glucanase in the protein extract was determined according to Kini et al. (2000) with minor modifications. Samples containing 20 µg of protein were incubated with a mixture of 0.1% (w/v) laminarin in a 0.05 M acetate buffer (pH 5.2). The mixture was incubated for 15 min at 37 °C in a thermal block (BIOER, CHB-202). The reaction was stopped by adding DNS reagent (0.5% (w/v) 3,5-dinitrosalicylic acid and 15% (w/v) sodium potassium tartrate tetrahydrate), and then, the samples were incubated for 15 min at 98 °C. The absorbance of samples was measured using a spectrophotometer (Ray-Leigh UV-2601 Spectrophotometer) at 540 nm. A standard glucose curve was used to calculate β−1,3-glucanase activity.

### Determination of total polyphenol content

Total polyphenolic content was determined according to Márquez-García (2012a). Two hundred mg of plant tissue was homogenized in 2 mL of 80% methanol and incubated for 2 h at 4 °C. The samples were centrifuged at 10,000 × g for 10 min. One hundred microlitres of the obtained supernatant was added to the reaction mixture (3 mL distilled water, 0.2 mL Folin-Ciocalteu reagent, 0.6 mL 7.5% Na₂CO₃). The resulting mixture was vortexed and incubated for 2 h at room temperature (22 °C) in the dark. After incubation, the absorbance of the samples was measured at 765 nm (Ray-Leigh UV-2601 Spectrophotometer). Total polyphenol content in the samples was determined relative to the gallic acid standard.

### Statistical analyses

All parameters were measured in at least three repetitions. The obtained data was statistically processed using Canoco5 software (Ter Braak and Šmilauer 2012). The normality and intact distribution of data distribution were tested using the Shapiro–Wilk (SW) test; then, a parametric multifactorial ANOVA was used to test differences in tested parameters based on genotype/variety (Aragon, Bay Yan 2, Ivory, Racoon, and Vaclav) and experimental variant (C, Cd, P, and P + Cd). Differences between individual experimental variants for each measured parameter were tested by one-factor ANOVA. The significance of differences was expressed at three probability levels: *p* < 0.05, *p* < 0.01, and *p* < 0.001. The correlation between metal content in tissues and some parameters was evaluated (Pearson).

## Results

No visual symptoms of toxicity were observed as a result of the applied dose of Cd (Suppl. Figure 1). As a consequence of plant infection by powdery mildew, powdery leaves were visibly present within a few days in all oat varieties (Suppl. Figure 1).

### Photosynthetic pigment content

**Fig. 2:**
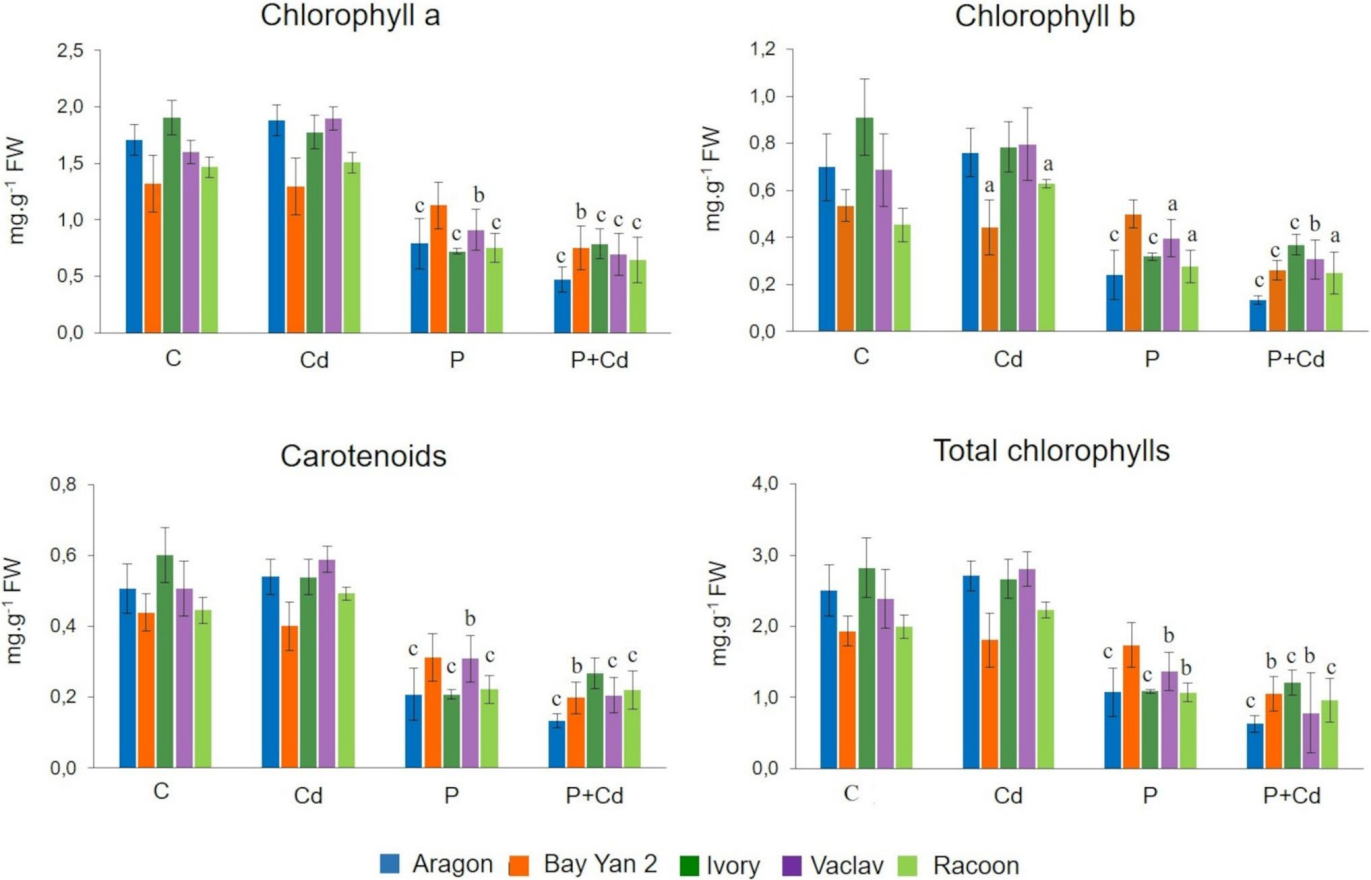
Photosynthetic pigment content in the leaves of five oat varieties exposed to cadmium (Cd), pathogen (P, *Blumeria graminis*), and their combination (P + Cd). C, control; FW, fresh weight. Data represent the arithmetic mean ± standard deviation (*n* = 3). Statistically significant changes at levels: a (*p* < 0.05), b (*p* < 0.01), and c (*p* < 0.001) compared to the control (one-way ANOVA, Dunnett’s test)

The tested oat varieties exhibited genotypic variability in the content of photosynthetic pigments in response to Cd, as well as to P and P + Cd treatments (Fig. 2).

In untreated leaves, the content of Chl*a* ranged from 1.41 to 1.84 mg·g⁻^1^ FW, Chl*b* from 0.45 to 0.87 mg·g⁻^1^ FW, carotenoids from 0.45 to 0.58 mg·g⁻^1^ FW, and total chlorophylls from 1.96 to 2.82 mg·g⁻^1^ FW. Cd treatment did not cause significant changes in the content of photosynthetic pigments, with the exception of var. Racoon, in which Chl*b* significantly increased to 0.63 mg·g⁻^1^ FW (Fig. 2).

A markedly lower pigment content was recorded in infected leaves. Chl*a* levels ranged from 0.72 to 1.17 mg·g⁻^1^ FW in the P variant and from 0.47 to 0.84 mg·g⁻^1^ FW in the P + Cd variant. A similar trend was observed for Chl*b* (0.24–0.50 mg·g⁻^1^ FW in P; 0.13–0.37 mg·g⁻^1^ FW in P + Cd), carotenoids (0.19–0.32 mg·g⁻^1^ FW in P; 0.13–0.26 mg·g⁻^1^ FW in P + Cd), and total chlorophylls (1.07–1.74 mg·g⁻^1^ FW in P; 0.63–1.24 mg·g⁻^1^ FW in P + Cd). All changes in the infected treatments were statistically significant, with the exception of var. Bay Yan 2, where a significant decrease in total chlorophyll content (by 46%) was observed only in the P + Cd variant (Fig. 2).

### Cadmium and calcium content in plant tissues

Different oat vars. accumulated varying amounts of Cd and Ca in oat tissues. The measured Cd content in the shoots of control plants ranged from 0.24 to 1.01 mg.kg⁻^1^ DW (controls), while in the shoots of the Cd variant, it ranged from 13.72 to 35.31 mg.kg⁻^1^ DW (Table 1). In the shoots of infected plants (P + Cd variant), Cd accumulation ranged from 48.17 to 96.20 mg.kg⁻^1^ DW, which is on average 3.55 times higher than in the Cd variant (Table 1). The Racoon var. accumulated the most Cd in shoots within the Cd variant (35.31 mg.kg⁻^1^ DW), and the Ivory var. accumulated the highest Cd content within the P + Cd variant (96.20 mg.kg⁻^1^ DW) (Table 1). Roots accumulated more Cd than shoots (0.60–1.35 mg·kg⁻^1^ DW in the C variant, 63.52–308.85 mg·kg⁻^1^ DW in the P variant, and 151.00–214.67 mg·kg⁻^1^ DW in the P + Cd variant) (Table 1).

**Table 1:** Cadmium content in shoots and roots (mg.kg^−1^ dry matter)

	Control	Cd	P + Cd
	Shoot		
Aragon	0.31 ± 0.04	14.12 ± 0.83 a	60.07 ± 9.68 c
Bay Yan 2	0.42 ± 0.08	13.72 ± 0.28 c	70.53 ± 3.28 c
Ivory	0.24 ± 0.03	21.02 ± 1.23 b	96.20 ± 29.56 c
Vaclav	1.01 ± 0.69	28.48 ± 2.36 b	48.17 ± 12.84 c
Racoon	0.24 ± 0.03	35.31 ± 0.04 a	75.83 ± 23.84 c
	Root		
Aragon	1.35 ± 0.10	63.52 ± 4.30 c	167.33 ± 2.52 c
Bay Yan 2	0.60 ± 0.07	81.42 ± 5.01 c	185.00 ± 2.00 c
Ivory	0.82 ± 0.07	281.66 ± 26.95 c	307.33 ± 2.08 c
Vaclav	0.63 ± 0.09	164.18 ± 8.16 c	151.00 ± 1.00 c
Racoon	0.91 ± 0.05	308.85 ± 16.71 c	214.67 ± 1.53 c

Data is presented as means ± standard deviation (*n* = 3). Cd (cadmium), P (pathogen, *Blumeria graminis*), P + Cd (pathogen + cadmium). Lowercase letters (a, b, and c) indicate significant differences between control and treatment (one-way ANOVA, Dunnett’s test) at *p* < 0.05 (a), *p* < 0.01 (b), and *p* < 0.001 (c)

**Fig. 3:**
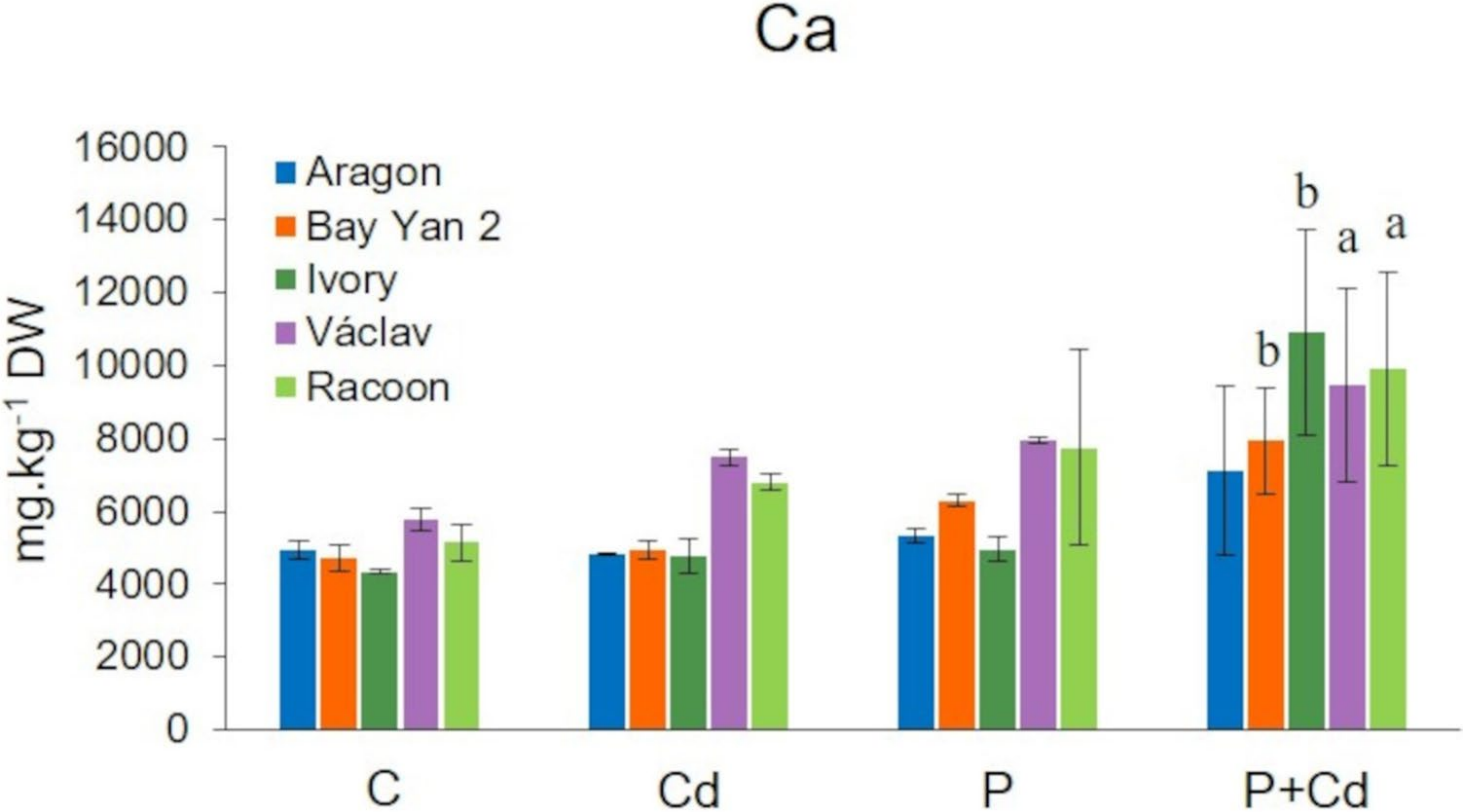
Calcium (Ca) content in the leaves of five oat varieties. DW, dry weight. Data represents the arithmetic mean ± standard deviation (*n* = 3). Statistically significant changes at levels a (*p* < 0.05) and b (*p* < 0.01) compared to the control. C (control), Cd (cadmium), P (pathogen, *Blumeria graminis*), P + Cd (pathogen + cadmium) (one way ANOVA, Dunnett’s test)

The tested vars. showed differences in basal Ca levels, with the highest Ca content found in the leaves of the Vaclav and Racoon vars. (5755 mg·kg⁻^1^ and 5145 mg·kg⁻^1^, respectively) and the lowest in var. Ivory (4350 mg·kg⁻^1^) (Fig. 3). This pattern was more or less maintained in the stressed plants. The Ca content generally increased in all vars. and in all experimental variants in the order: C < Cd < P < P + Cd. Due to Cd and powdery mildew, the Ca content in the leaves increased non-significantly to 4765–7495 mg·kg⁻^1^ and 4951–7946 mg·kg⁻^1^, respectively, with the highest content found in the Ivory, Vaclav, and Racoon vars. (Fig. 3). Statistically significant increases in Ca content were observed only in the infected leaves of the Bay Yan 2 (7934 mg.kg⁻^1^), Ivory (10,910 mg.kg⁻^1^), Vaclav (9455 mg.kg⁻^1^), and Racoon (9903 mg.kg⁻^1^) vars. under combined stress (Fig. 3).

### Lipid peroxidation of membranes

**Fig. 4:**
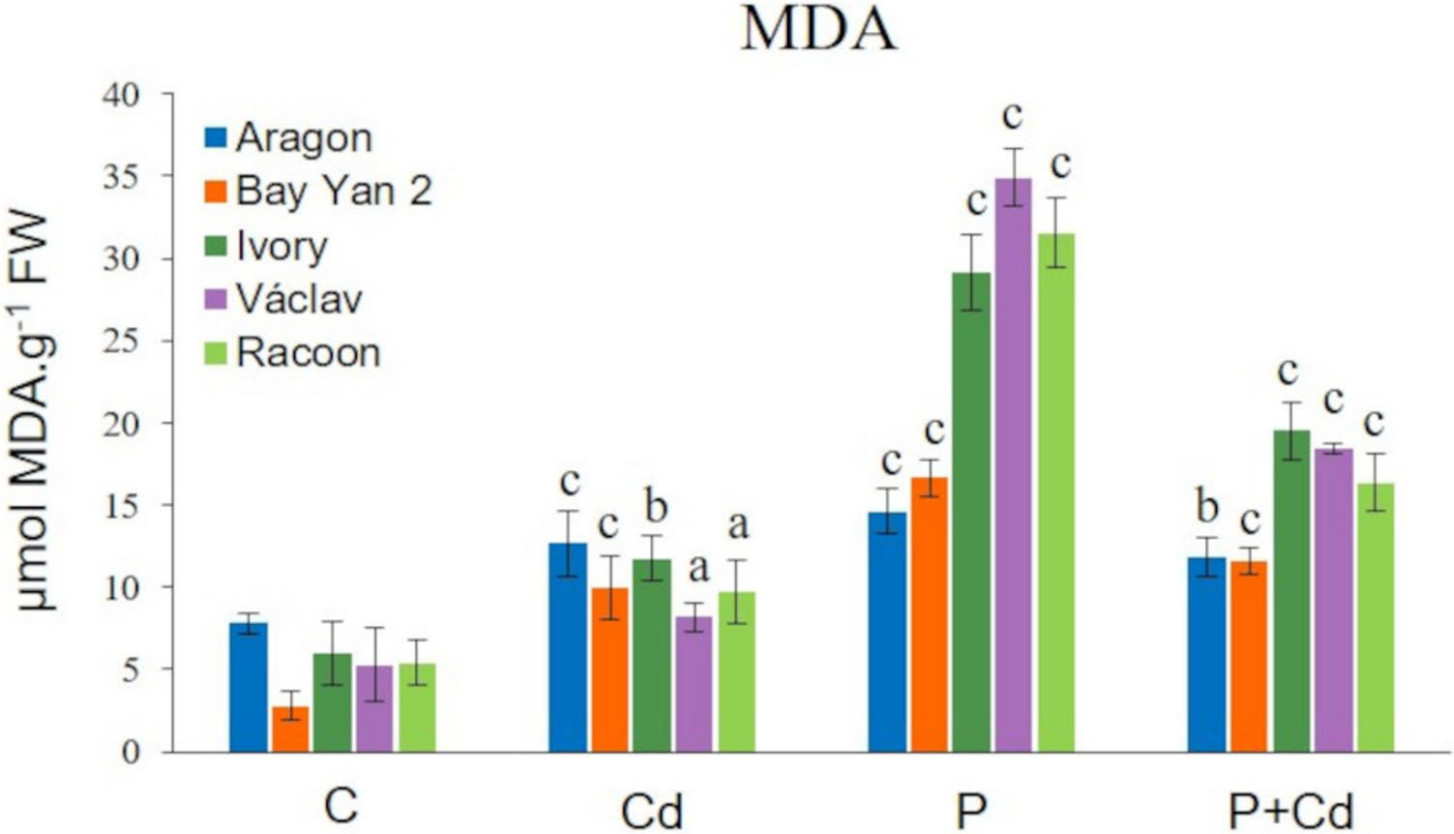
Changes in malondialdehyde (MDA) content in the leaves of five oat varieties due to cadmium (Cd), pathogen (P, *Blumeria graminis*), and their combination (P + Cd). C, control; FW, fresh weight. Data represent the arithmetic mean ± standard deviation (*n* = 4). Statistically significant changes at levels a (*p* < 0.05), b (*p* < 0.01), and c (*p* < 0.001) compared to control (one-way ANOVA, Dunnett’s test)

The MDA content in plant tissues indirectly indicates the degree of damage to cell membranes under stress conditions. The basal MDA level in leaves of untreated plants ranged from 2.80 to 7.80 µmol·g⁻^1^, with the lowest level observed in var. Bay Yan 2 and the highest in var. Aragon (Fig. 4). MDA content significantly increased in the leaves of all oat vars. and in all experimental treatments in relation to the control (Fig. 4). The most severe membrane damage (MDA 14.63–34.92 µmol·g⁻^1^) was observed in the infected leaves (P variant), while less membrane damage (MDA 11.55–19.53 µmol·g⁻^1^) was detected in the leaves exposed to combined stress (P + Cd variant). The least, yet still significant, damage occurred in leaves exposed to cadmium alone (MDA 8.19–12.71 µmol·g⁻^1^) (Fig. 4).

### Catalase activity

**Fig. 5:**
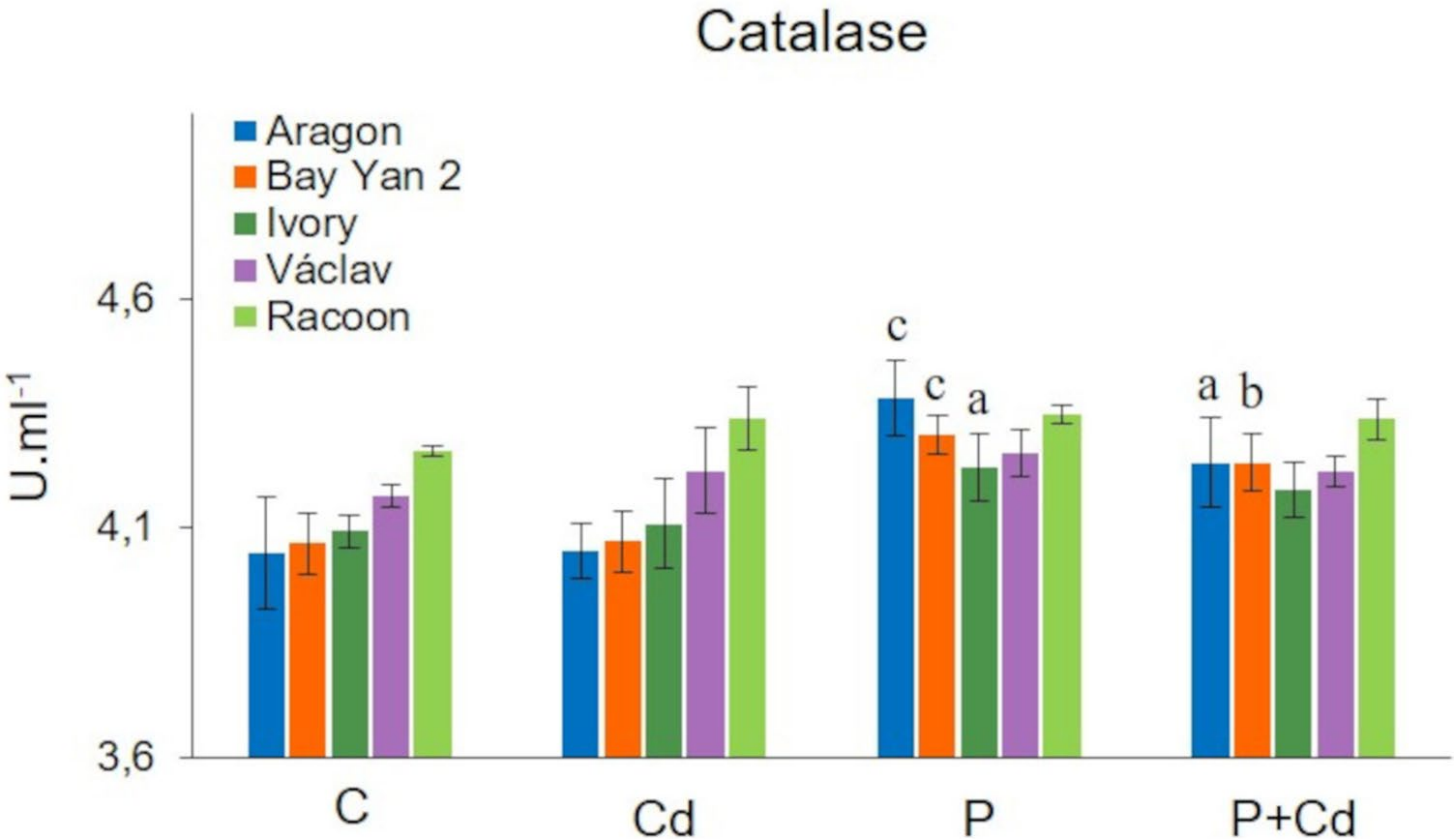
Changes in catalase activity in the leaves of five oat varieties due to cadmium (Cd), pathogen (P, *Blumeria graminis*), and their combination (P + Cd). C, control. Data represents the arithmetic mean ± standard deviation (*n* = 4). Statistically significant changes at levels a (*p* < 0.05), b (*p* < 0.01), and c (*p* < 0.001) compared to control (one-way ANOVA, Dunnett’s test)

CAT is an antioxidant enzyme, with one unit (U) defined as the amount that decomposes 1.0 µmol of H₂O₂ per minute at pH 7 and 25 °C. The evaluated oat vars. differed in their basal CAT activity, with the highest activity observed in the leaves of var. Racoon (4.27 U.mL^−1^) and the lowest in var. Aragon (4.05 U.mL^−1^) (Fig. 5). A statistically significant increase in CAT activity was recorded in the infected leaves (P variant) of vars. Aragon, Bay Yan 2, and Ivory (4.38, 4.30, and 4.23 U.mL^−1^, respectively). Additionally, under combined stress (P + Cd), CAT activity increased in the leaves of Aragon and Bay Yan 2, reaching 4.24 U·mL⁻^1^ in both vars. Cadmium alone had almost no effect on CAT activity (Fig. 5).

### Glutathione content

**Fig. 6:**
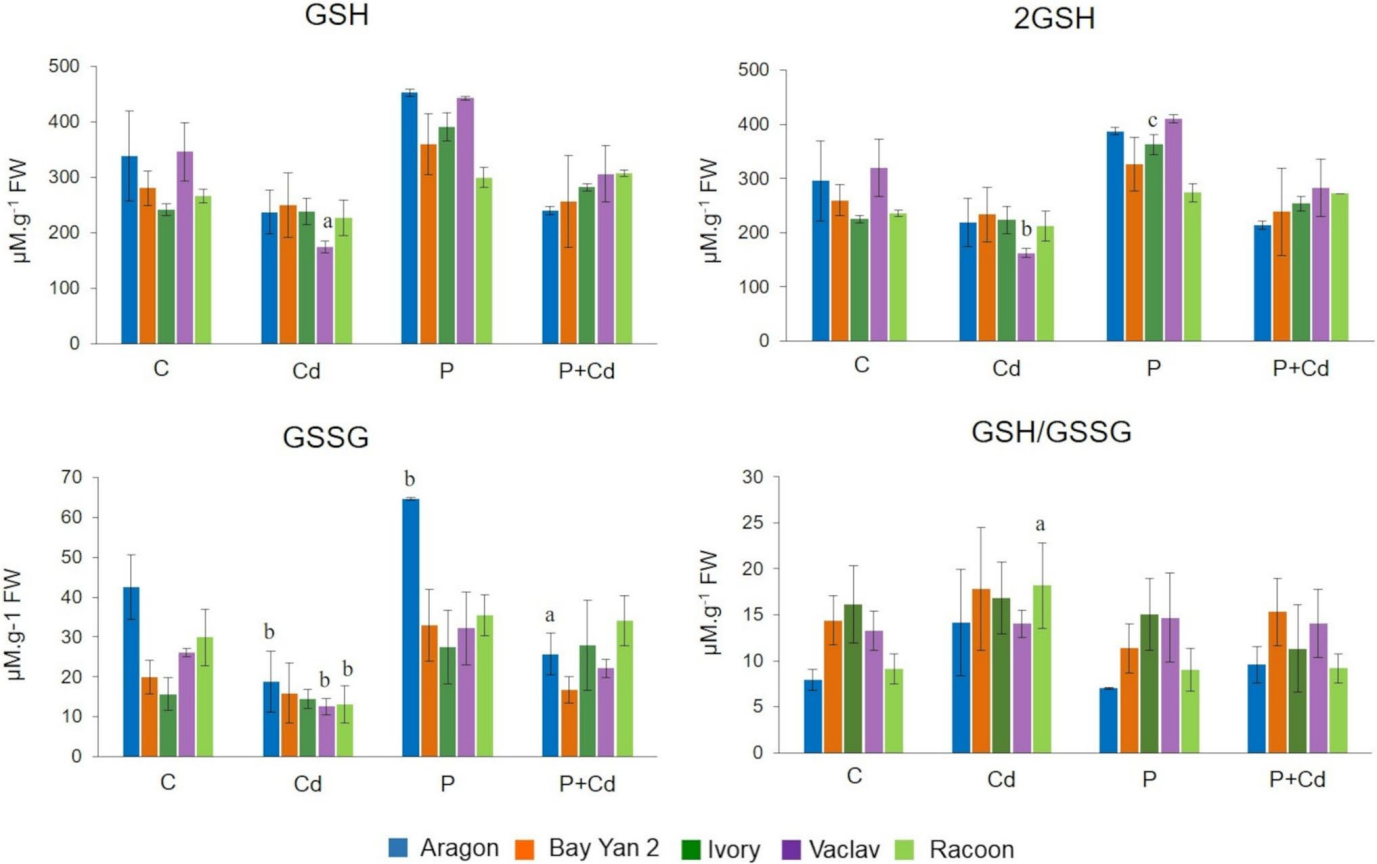
Changes in glutathione content in the leaves of five oat varieties exposed to cadmium (Cd), pathogen (P, *Blumeria graminis*), and their combination (P + Cd). C (control), GSH (total glutathione), 2GSH (reduced glutathione), GSSG (oxidized glutathione), GSH/GSSG ratio (the redox status), FW (fresh weight). The data represents the arithmetic mean ± standard deviation (*n* = 3). Statistically significant changes at levels a (*p* < 0.05), b (*p* < 0.01), and c (*p* < 0.001) compared to the control (one-way ANOVA, Dunnett’s test)

Glutathione plays a significant role in plant defence against various stressors. In the control (C) variant, the individual oat vars. contained varying levels of GSH (241.00–391.84 µM·g⁻^1^ FW), 2GSH (225.36–365.76 µM·g⁻^1^ FW), and GSSG (15.65–42.48 µM·g⁻^1^ FW). The GSH/GSSG ratio ranged from 7.97 to 16.14 (Fig. 6). In the Cd variant, a general decrease in all forms of glutathione was observed compared to the control, with a significant decrease in 2GSH in the leaves of var. Vaclav (222.05 µM·g⁻^1^ FW) and in GSSG in the vars. Aragon (18.84 µM·g⁻^1^ FW), Vaclav (12.62 µM·g⁻^1^ FW), and Racoon (13.15 µM·g⁻^1^ FW) (Fig. 6).

In the P variant, all forms of glutathione increased, with a significant rise in GSH levels in vars. Ivory (390.69 µM·g⁻^1^ FW), Vaclav (442.41 µM·g⁻^1^ FW), and Aragon (452.12 µM·g⁻^1^ FW). However, 2GSH levels were lower in the leaves of var. Ivory (363.12 µM·g⁻^1^ FW), while GSSG levels were elevated in var. Aragon (64.62 µM·g⁻^1^ FW) (Fig. 6).

Under combined stress (P + Cd), no significant changes in total glutathione content were detected compared to the control, except for a significant increase in GSSG in the leaves of var. Aragon (25.73 µM·g⁻^1^ FW) (Fig. 6). The cellular redox status, expressed as the GSH/GSSG ratio, remained largely unchanged under stress conditions, with a significant increase observed only in var. Racoon (9.19) (Fig. 6).

### Total polyphenol content

**Fig. 7:**
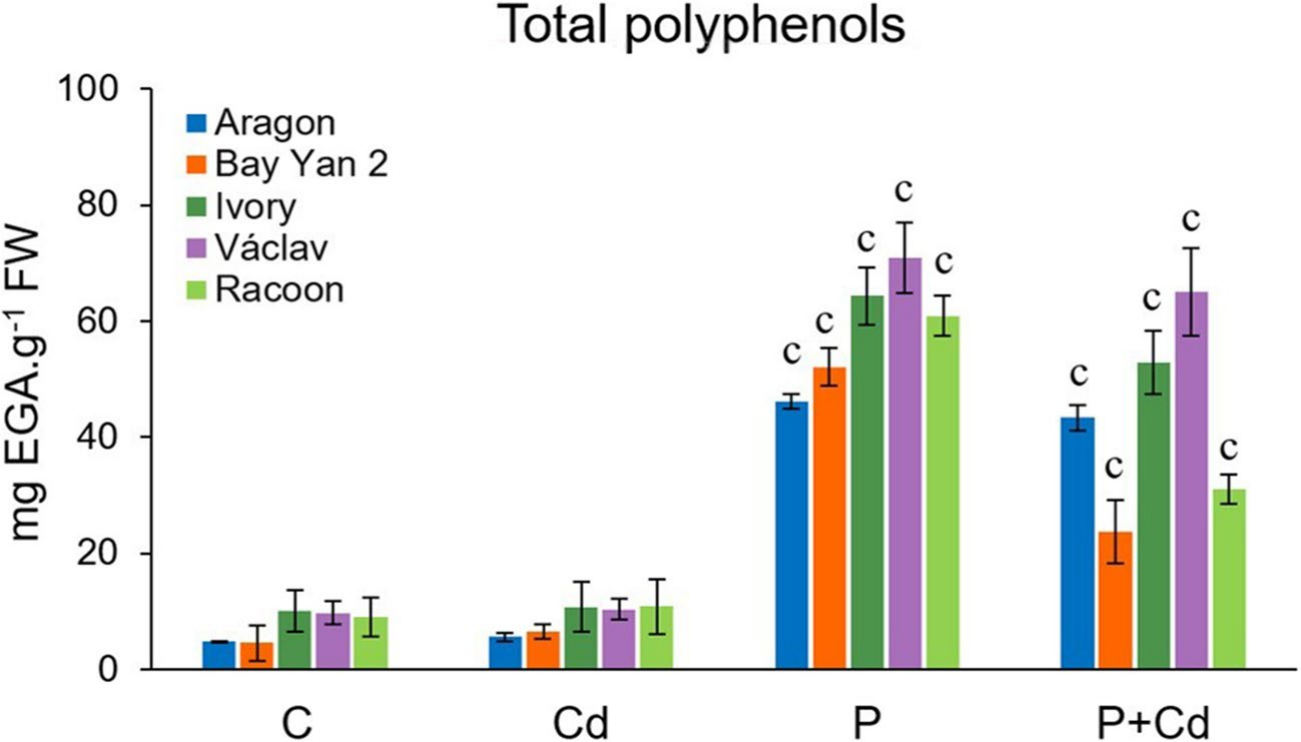
Total polyphenol content in the leaves of five oat varieties exposed to cadmium (Cd), pathogen (P, *Blumeria graminis*), and their combination (P + Cd). C (control), EGA (gallic acid concentration equivalent), FW (fresh weight). The data represents the arithmetic mean ± standard deviation (*n* = 4). Statistically significant changes at level c (*p* < 0.001) compared to the control (one-way ANOVA, Dunnett’s test)

The leaves of control plants contained 4.53–9.70 mg·g⁻^1^ of total polyphenols, depending on the variety (Fig. 7). Cd exposure caused a slight, statistically non-significant increase in polyphenol content compared to control (5.57–10.81 mg·g⁻^1^). In infected leaves, polyphenol levels were Markedly elevated, reaching 46.14–70.91 mg·g⁻^1^ in the P variant and 23.68–65.07 mg·g⁻^1^ in the P + Cd variant, with the highest content recorded in the leaves of var. Vaclav (Fig. 7).

### Activity of total β−1,3-glucanases

**Fig. 8:**
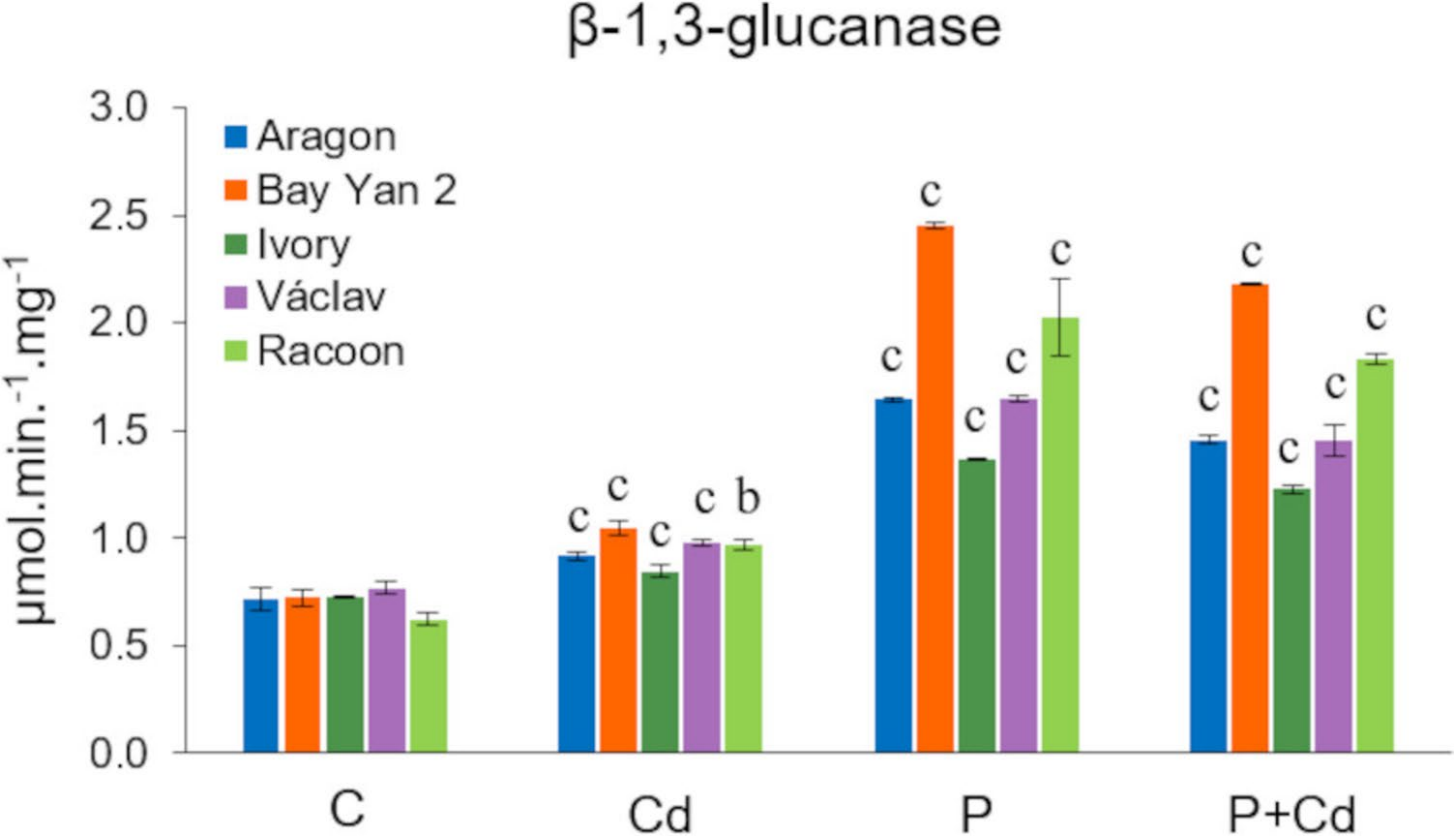
Changes in the activity of total β−1,3-glucanases in the tested varieties of oat. C (control), Cd (cadmium), P (pathogen, *Blumeria graminis*), P + Cd (pathogen + cadmium). The data represents the arithmetic mean ± standard deviation (*n* = 3). Statistically significant changes at levels b (*p* < 0.01) and c (*p* < 0.001) compared to the control (one-way ANOVA, Dunnett’s test)

The individual oat cultivars did not show significant differences in basal glucanase activity (0.72–0.77 µmol·min⁻^1^·mg⁻^1^). Exposure to stress led to a significant increase in enzyme activity, with the lowest values observed in the Cd variant (0.85–0.98 µmol·min⁻^1^·mg⁻^1^). Infected leaves of all vars. showed Markedly higher activity, reaching up to 2.45 µmol·min⁻^1^·mg⁻^1^ in the P variant and up to 2.18 µmol·min⁻^1^·mg⁻^1^ in the P + Cd variant. The highest activity was detected in var. Bay Yan 2, while the lowest was recorded in var. Ivory (Fig. 8).

### Results of two-factor ANOVA

The two-factor ANOVA revealed significant differences between the variants and genotypes for most of the tested parameters. An interaction between the genotype and experimental variant was also confirmed (Table S1). A more detailed comparison of the variants across individual parameters is provided by the results of the one-factor ANOVA (Table S2). The P and Cd variants differed significantly in almost all monitored parameters in most of the tested oat cultivars (with the exception of Ca content, CAT activity, and GSH/GSSG ratio). A similar pattern was observed for the Cd and P + Cd variants, which did not differ significantly in Ca content, CAT activity, and glutathione content in most cultivars. In contrast, the P and P + Cd variants differed significantly in MDA content, GSH, 2GSH, polyphenol levels, and glucanase activity (Table S2).

## Discussion

The goal of this study was to understand the mechanisms that contribute to oat tolerance to Cd ions, the pathogen *Blumeria graminis* f. sp. *avenae*, and their combined effects. We additionally evaluated the potential priming effect of Cd on plants exposed to powdery mildew.

### Cd accumulation and Cd tolerance

**Fig. 9:**
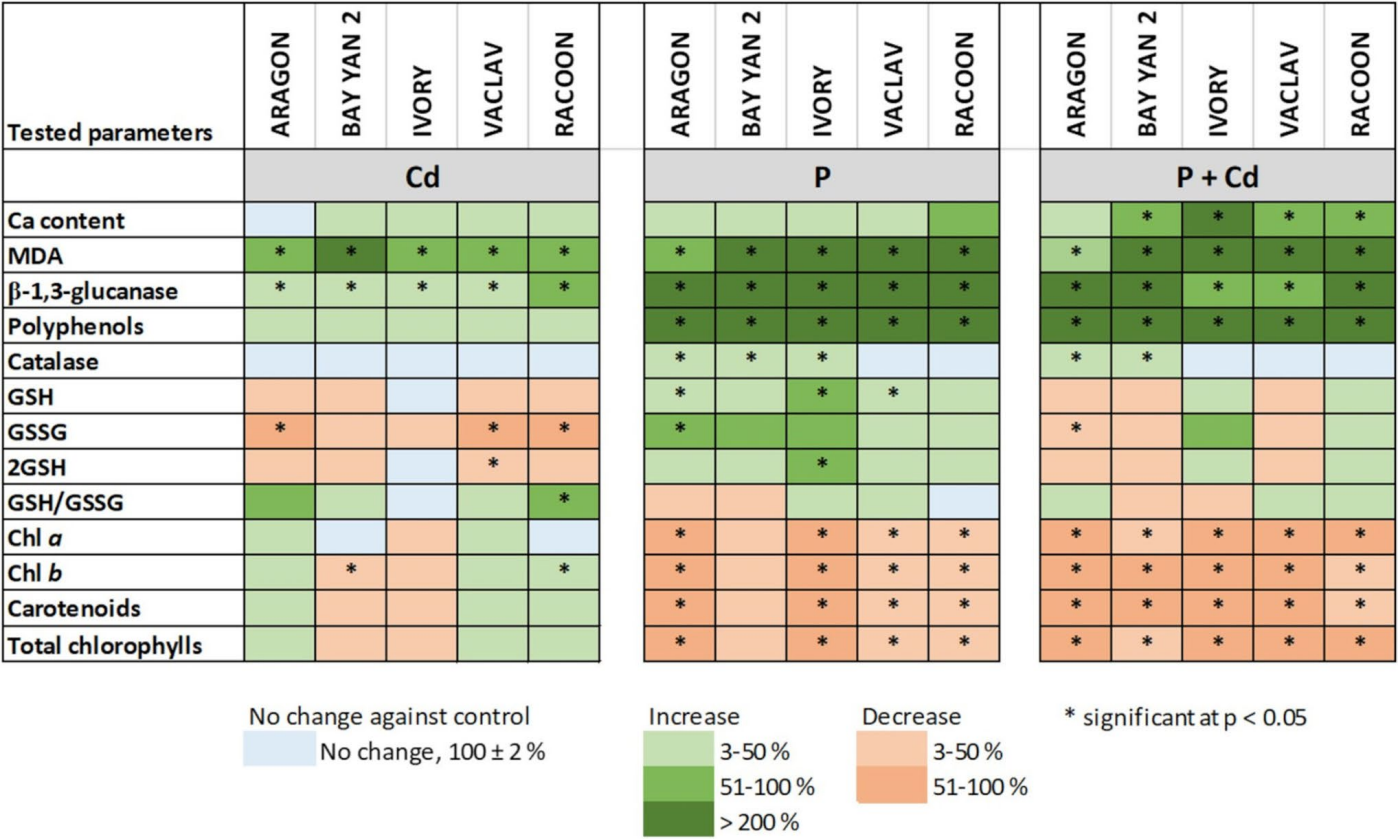
The effect of cadmium (Cd) and pathogen (P, *Blumeria graminis*) applied individually and in combination on parameters estimated in shoots/leaves: calcium (Ca) content, malondialdehyde (MDA) content, activity of β−1,3-glucanase, activity of catalase, content of glutathione (GSH, total; 2GSH, reduced; GSSG, oxidised), ratio of total and oxidized glutathione (GSH/GSSG), photosynthetic pigment content (Chl*a*, chlorophyll *a*; Chl*b*, chlorophyll *b*, total chlorophylls and carotenoids). *Statistically significant changes

In this study, oats exhibited high tolerance to the Cd dose (50 mg.kg^−1^ of soil). We expected this response to the chosen Cd dose, as previous pilot testing on different doses of Cd (0–400 mg.kg^−1^ of soil) in oats had suggested high tolerance even at the highest tested dose (Kubová et al. 2023). Therefore, the choice of Cd dose was not straightforward. High Cd doses (above 50 mg.kg^−1^ of soil) are typical only for localized soil contamination (which is also rare). When selecting lower doses (< 50 mg.kg^−1^), we did not expect significant changes in most of the measured parameters. Moreover, we needed to use a lower metal dose where a “priming effect” on the plants was expected. The chosen Cd dose was also based on existing studies on oat tolerance to Cd ions (discussed below). Oat plants exposed to Cd for 42 days did not show any visible symptoms of toxicity (Suppl. Figure 1), and we observed almost no inhibitory effect on the content of photosynthetic pigments (Figs. 2 and 9). There is relatively little data on evaluating oat tolerance to Cd ions, and the results are controversial. High tolerance to Cd up to 20 mg.kg^−1^ soil was pointed out by, e.g. Ciecko et al. (2004), Uraguchi et al. (2009), and Tůma et al. (2014). Rolka (2015) also noted an overall stimulating effect on the growth of *Avena sativa* L., cv. Dragon due to a dose of 40 mg.kg^−1^. On the other hand, growth inhibition, a decrease in photosynthetic pigment content, and yield reductions were recorded even at a dose of 10 mg.kg^−1^ of soil (Boros-Lajszner et al. 2019).

The amount of Cd accumulated in the tissues of the plants depended on genotype, with the plants preferentially accumulating Cd in the roots being consistent with other studies (Tůma et al. 2014; Boros-Lajszner et al. 2019). For all oat varieties, we observed a significantly higher accumulation of Cd in the leaves of infected variants of the experiment (Table 1, Table S2). The higher accumulation of Cd in the shoots of infected plants is likely due to the failure of defence mechanisms that usually prevent Cd transport to the upper parts of the plant. Oats accumulated relatively high amounts of Cd in the roots, comparable to the amounts of Cd accumulated in the *Avena sativa* cv. Moozart at a dose of 53.61 mg.kg^−1^ of soil (Azizian et al. 2011). However, Tůma et al. (2014) reported 11.37 mg.kg^−1^ of Cd in younger leaves, 55.97 mg.kg^−1^ in older leaves, and 235.33 mg.kg^−1^ in roots at a dose of 20 mg.kg^−1^ of soil. On the other hand, black oats (*Avena strigosa*, Schreb.) accumulate and detoxify more Cd in the shoots than in other parts of the plant (Uraguchi et al. 2006).

### Effect of Cd, *Blumeria graminis*, and their combined action on physiological and biochemical parameters

#### Photosynthetic pigment content

The content of photosynthetic pigments was significantly reduced due to infection (P), and also due to the combination of P + Cd. Cd alone had almost no significant effect on pigment content despite the relatively high accumulation of Cd in the leaves (Figs. 2 and 9). These changes confirm the high tolerance of the tested genotypes to Cd, but their low tolerance to the tested pathogen. Previous studies evaluating the effect of Cd on photosynthetic activity show a decrease in the content of photosynthetic pigments in leaves (Zhao 2011; Kumar et al. 2019). However, findings of increased photosynthetic activity due to certain doses of Cd are not rare. Kumar and Dwivedi (2018), for example, recorded an increased total chlorophyll content in response to a dose of 11.24 mg Cd.L^−1^ (100 µM). It is also stated that low concentrations of Cd (0.005 and 0.01 mg.L^−1^, 0.05 and 0.1 µM) have a stimulatory effect on chlorophyll synthesis and photosynthetic activity (Siddique et al. 2018). The decrease in the content of photosynthetic pigments as a result of infection by powdery mildew is consistent with other authors’ findings (Akhkha et al. 2003; Saja et al. 2020). The penetration of pathogen hyphae into the leaf tissues often leads to severe damage to the photosynthetic apparatus and chlorophyll breakdown (Akhkha et al. 2003; Saja et al. 2020).

#### Calcium content

Calcium is a fundamental macronutrient and also a signalling molecule that participates in various physiological processes in plants, such as growth and development and photosynthesis (Gupta et al. 2023). Due to the chemical similarity between Ca and Cd (similar charge and ionic radius), Ca can also mediate Cd-induced physiological or metabolic changes in plants (Perfus-Barbeoch et al. 2002). In our experiments, there was generally an increase in the Ca content in the tissues of oats, with a significant rise in the leaves of Bay Yan 2, Vaclav, and Racoon vars. exposed to both Cd and powdery mildew (Figs. 3 and 9). The Ca content positively correlated with the Cd content in the roots (*R* = 0.73 for the Cd variant and *R* = 0.82 for the P + Cd variant) and in the leaves (*R* = 0.46 for the Cd variant and *R* = 0.81 for the P + Cd variant). Increased Ca content in the tissues of Cd-treated plants has also been reported by other authors (Tahjib-Ul-Arif et al. 2021; Gupta et al. 2023). Recent studies have shown that the protective effect of Ca under Cd stress involves alleviating growth inhibition, regulating metal uptake and translocation, improving photosynthesis, mitigating oxidative damage through activation of antioxidant enzymes, and also inducing the synthesis of callose, proline, pectin, and defence proteins (Hayat et al. 2007; Islam et al. 2010; Steinhorst and Kudla 2014; Huang et al. 2017). A similar role is played by Ca in plant defence against pathogens (Jiang and Ding 2023), with the significant mechanism being callose formation mediated by Ca. Callose (β−1,3-glucan) is rapidly synthesized at sites of tissue damage to mechanically prevent further pathogen penetration (Wang et al. 2021). A similar role can be played in tissues exposed to heavy metals (Li et al. 2023). In the case of Cd, its accumulation can be observed only at higher doses of the metal (Piršelová et al. 2012). Several studies have also pointed out that Ca reduces Cd uptake by roots while increasing its transport to shoots, which leads to changes in the remediation potential of plants (Li et al. 2016; Ye et al. 2020). Ca also significantly contributes to plant-pathogen interactions, and its role in the induction of PR-protein synthesis has been confirmed (Ghorbel et al. 2021). The role of Ca in defence against Cd as well as pathogens is therefore essential. As a signaling molecule, Ca plays a central role in the rapid activation of plant defence responses by initiating signaling cascades that lead to the expression of defence-related genes and the induction of antioxidant enzymes. Under stress conditions induced by Cd or pathogens, cytosolic Ca levels increase, activating Ca-dependent protein kinases (CDPKs) and other regulatory proteins (Bhar et al. 2023). These pathways enhance the activity of key antioxidant enzymes such as superoxide dismutase (SOD), CAT, and ascorbate peroxidase (APX) which help mitigate oxidative stress by scavenging excessive ROS. Moreover, Ca contributes to the stabilization of cellular membranes and reduces heavy metal toxicity, maintaining cellular integrity. Importantly, Ca also interacts with plant hormone signaling networks, thereby modulating hormonal balance and amplifying stress-responsive pathways. Through these combined effects, Ca reinforces both local and systemic resistance, enhancing the plant’s overall adaptability and resilience under stress (Naz et al. 2024).

#### Detection of oxidative stress and mechanisms of its elimination (catalase activity, glutathione content)

##### Lipid peroxidation of membranes

Plants respond to various forms of stress with increased accumulation of reactive oxygen species, leading to oxidative stress. The degree of oxidative damage can be indirectly detected by the accumulation of MDA—a product of lipid peroxidation in cellular membranes (Montillet et al. 2004; Gratão et al. 2005). Although no major changes in photosynthetic pigment content were observed in Cd-treated plants, MDA content increased significantly (1.54–3.57 times) compared to control samples (Figs. 4 and 9). Higher levels of membrane lipid peroxidation were also observed in infected leaves (1.88–6.59 times the control) which is consistent with the study by Ma et al. (2025). That study also confirmed genotypic differences in the level of MDA production in oat leaves infected with powdery mildew. However, membrane peroxidation was partially suppressed in the leaves of the P + Cd variant compared to the P variant (Fig. 4). This reduction in oxidative damage in this case can be attributed to mechanisms that likely enhance defence due to Cd exposure (the priming effect). These mechanisms may also involve increased accumulation of Ca^2+^, polyphenols, PR-proteins, or GSH-induced glutathionylation, which protects proteins against oxidative damage (Mohapatra and Mittra 2017). The priming effect in the case of metal exposure to plants is especially noticeable at concentrations not excessively toxic to the plant (Liu et al. 2022). Effective defence mechanisms in the P + Cd variant are evidenced by the fact that we recorded lower MDA levels (compared to P), despite higher Cd accumulation in tissues compared to the Cd variant. Similar conclusions were reached by Jali et al. (2019), who observed a 40% increase in MDA content in rice leaves due to Cd, a 61% increase in leaves infected with the fungus *Pyricularia oryzae*, and a 30% increase in leaves exposed to combined stress. A higher degree of lipid membrane peroxidation due to Cd and powdery mildew was also observed in the leaves of other crops (Tamás et al. 2008; Zhao 2011; Szalai et al. 2020; Zhang and Gao 2021).

##### Catalase activity

Plants respond to increased oxidative stress with an antioxidant system, which includes antioxidant enzymes (SOD, CAT, and other related enzymes) and various non-enzymatic molecules (vitamins, polyphenols, glutathione, etc.). The role of this system is to eliminate oxidative stress. It has been demonstrated that CAT genes are essential for regulating plant responses to various abiotic and biotic stressors, with many members of the *CAT* gene family exhibiting unique spatiotemporal expression patterns and responding differently to environmental and developmental stimuli (Ghorbel et al. 2023; Liu et al. 2023). No significant changes in CAT activity were observed in the Cd variant, while variants with infection showed a slight (statistically significant in some varieties) increase in this enzyme’s activity (Figs. 5 and 9). Although an increase in CAT activity due to Cd is a commonly observed phenomenon (Baker et al. 2023), a decrease in its activity has also been recorded (Patel et al. 2013). Previous studies suggest that CAT activity in tissues of Cd-treated plants is strongly dependent on metal dose and genotype (Shah et al. 2001; Ghorbel et al. 2025). Genotypic variability in CAT activity was also recorded in infected variants of the experiment (a statistically significant increase was observed in Aragon, Bay Yan 2, and Ivory vars., Fig. 9). Increased CAT activity was also observed in the leaves of oats (Ghorbel et al. 2023), chickpeas (Narayan et al. 2017), castor beans (Bharathi et al. 2019), and barley (Singla et al. 2019) exposed to various pathogens.

So far, ten *AvCAT* genes have been identified in *Avena sativa*, which are classified into three groups (Groups I–III) (Ghorbel et al. 2023). In terms of response to various environmental stressors, *AvCAT1*, *AvCAT2*, *AvCAT4*, and *AvCAT8* showed the most notable activity (Ghorbel et al. 2023, 2025). Some of these genes, such as *AvCAT1*, are strongly expressed in leaves under normal conditions but only weakly in roots. Under heavy metal stress, the *AvCAT1* gene was upregulated in the presence of 50 µM AlCl₃, CdCl₂, and CuCl₂. In fact, CdCl₂-induced stress triggered a rapid activation of *AvCAT1* in roots; however, no significant changes in *AvCAT1* transcript levels were observed in the leaves (Ghorbel et al. 2025).

##### Glutathione content

Glutathione is a sulphur-containing compound that plays several important roles in the tissues of organisms (Vašková et al. 2023). Metals affect glutathione metabolism mainly at two levels: (a) they increase GSH oxidation and the formation of phytochelatins (PC), resulting in decreased glutathione levels, and (b) they induce de novo synthesis of glutathione (Jozefczak et al. 2012). In the leaves of almost all oat varieties in the Cd and P + Cd variants, a decrease in glutathione levels was observed compared to the control (Figs. 6 and 9), while in the P variant, an increase in glutathione levels was recorded (with the most pronounced change in the Ivory var., Fig. 9). The 2GSH/GSSG ratio did not change significantly in any of the experimental variants (with the exception of a significant increase in the Racoon var. leaves) (Figs. 6 and 9). These results suggest that glutathione plays a different role in defence in the Cd and pathogen variants. This is also supported by the results of one-factor ANOVA tests, which confirmed a statistically significant difference between the Cd and P variants and between the P and P + Cd variants in most of the tested genotypes (Table S2). The decrease in glutathione due to Cd is likely the result of its preferential consumption as a precursor for the synthesis of PC—proteins involved in Cd detoxification. Its antioxidant role in these variants is insignificant due to minimal changes in the GSH/GSSG ratio. This hypothesis is also confirmed by the generally lower levels of GSH, 2GSH, and GSSG in the leaves of the P + Cd variant compared to the P variant (Fig. 6).

Previous studies on the effects of Cd on glutathione metabolism are contradictory, as the level of glutathione in plant tissues results from the activation of various components of the ascorbate–glutathione cycle and is largely influenced by various factors such as genotype, stressor type, and dose (Hasanuzzaman et al. 2017; Hendrix et al. 2020). For example, several studies have observed a decrease in cellular GSH levels as well as the activity of GR in plants exposed to toxic levels of heavy metals (Tewari et al. 2006; Márquez-García et al. 2012b). Excessive PC production often leads to the severe depletion of GSH, preventing it from performing its other important functions in antioxidant defence and signalling. In this case, sulphur compound homeostasis is also disrupted to continuously maintain GSH levels (van Baelen et al. 1980). On the other hand, several studies have pointed out an increase in GSH levels in plants due to Cd (Ruegsegger et al. 1990; Bergmann and Rennenberg 1993). Masood et al. (2011) observed an increase in GSH content and GR activity in the leaves of *Brassica juncea L.* under Cd treatment (200 mg Cd.kg^−1^ soil), along with a decrease in the GSH/GSSG ratio, indicating preferential use of GSH for eliminating oxidative damage. Several studies have also pointed out the protective effects of exogenously applied GSH in mitigating metal/metalloid toxicity (Asgher et al. 2017; Tang et al. 2023) through various mechanisms, including reducing metal content in tissues (Chen et al. 2010).

Increased glutathione levels in oat tissues infected with powdery mildew are consistent with findings from other authors (Carver et al. 1991, 1996), and as mentioned, its role is likely more antioxidant. Increased GSH levels in plants exposed to various pathogens have also been observed in Arabidopsis (Großkinsky et al. 2012) and tomato (Kuźniak and Sklodowska 2004). It has been shown that fungal infections induce various components of the ascorbate–glutathione cycle (Fodor et al. 1997). Vanacker et al. (1998) reported that biotic stress caused by *Blumeria graminis* reduced ascorbate and dehydroascorbic acid content, but increased GSH levels in leaves of oat. That GSSG content also decreased suggests stress might simultaneously activate GR, recycling GSSG via NADPH. The reduced GSSG levels may also indicate that GSH is not being used to neutralize reactive oxygen species (suppressed conversion to GSSG), or that GSH synthesis is insufficient.

##### Polyphenol content

Polyphenols may play an important role in plant defence against Cd and pathogens (Goncharuk and Zagoskina 2023), and it has been shown that polyphenols primarily fulfil this defensive function under infection conditions (Figs. 7 and 9). The accumulation of polyphenols in plant tissues exposed to Cd depends on the metal dose and the plant’s tolerance to a certain level of the metal (Nobahar et al. 2021). Increased polyphenol content has been found in the shoots of barley (Dudjak et al. 2004), chamomile (Kováčik and Bačkor 2007), and marshmallow (Zoufan et al. 2020) exposed to Cd. We hypothesize that polyphenols do not significantly contribute to defence against Cd due to the high tolerance of oats to the given dose of the metal. However, infection led to a significant accumulation of these secondary metabolites in the leaves of oats. Previous studies have also pointed out the relationship between fungal infection and increased polyphenol synthesis in the host plant (Grand et al. 1987; Komarova and Davidovich 1997; Tischler et al. 2018). A cumulative effect on phenol content due to the combined action of Cd and the pathogen was observed in rice (Jali et al. 2019). Mayama et al. (1981) suggested that the rapid accumulation of phenols may lead to the effective isolation of the pathogen at the site of its entry, and the main roles of such phenols are antioxidant and antimicrobial. Strong evidence suggests that the esterification of phenols to cell wall materials (forming structures similar to lignin through crosslinking) is a common phenomenon in resistance (Fry 1986). However, the polyphenol content in the P + Cd variant was lower than in the P variant, statistically significantly in several vars. (Table S2), suggesting a possible priming effect of the metal under these conditions.

#### β−1,3-glucanase activity

Β−1,3-glucanases are enzymes from the PR protein group that greatly contribute to plant defence against pathogens (Perrot et al. 2022), although previous studies have indicated their accumulation in plant tissues as a result of heavy metals, drought, and other stressors (Békésiová et al. 2008; Lv et al. 2022). The activity of total glucanases significantly increased under stress, with the least pronounced (but significant) change in the Cd variant, showing higher activity in infected leaves across all vars. (Fig. 8). These results suggest that this enzyme participates in defence against both Cd and powdery mildew. However, previous studies have pointed out that the activity of some glucanase isoforms in plants exposed to Cd increases, while others decrease (Bardáčová et al. 2016). It is assumed that their role in defence against the metal is more complex; they may involve the universal induction of signalling pathways activated under stress conditions or specific functions related to defence against the metal itself or the pathogen (e.g. processes regulating the transport of metals or pathogens in tissues). The accumulation and degradation of callose (via β−1,3-glucanases) at the symplast level represent, for example, a rather effective mechanism for regulating the transport of not only toxic elements (e.g. heavy metals) but also various microorganisms (Blackman et al. 2014; O´Lexy et al. 2018). Increased accumulation of these enzymes has been demonstrated in oats and other cereals infected with powdery mildew or other pathogens (Fink et al. 1988; Frič and Huttová 1993; Mohammadizadeh-Heydari et al. 2024). The lower activity of β−1,3-glucanases in the leaves of most vars. exposed to combined stress compared to the P variant (Fig. 8, Table S2) suggests a possible priming effect of Cd. Although the Cd-induced defence described so far primarily involves increased accumulation of stress-related proteins targeting pathogens (Mittra et al. 2004; Morkunas et al. 2018), in our case, we rather assume an indirect effect of Cd, in the sense of inducing defence mechanisms aimed, for example, at reducing pathogenesis, where increased accumulation of PR proteins is not as urgent. A direct toxic effect of Cd on powdery mildew due to its increased accumulation in infected plants also cannot be ruled out. However, confirmation of these assumptions requires further in-depth research.

## Conclusions

The results of the analyses indicated different levels of toxicity from Cd and powdery mildew. While the applied Cd dose did not represent significant stress for oat plants despite increased membrane lipid peroxidation and β−1,3-glucanase activity, powdery mildew infection led to significant toxicity symptoms (sharp decrease in photosynthetic pigment content, high degree of membrane damage). Greater accumulation of polyphenols and Ca, along with higher activity of β−1,3-glucanases and CAT in infected leaves, suggests that these biomolecules are primarily involved in defence against pathogens. An interesting finding was that infected plants accumulated more Cd than non-infected plants. Nevertheless, in the P + Cd variant, we observed less membrane damage and lower polyphenol content, and also lower glucanase activity. This is likely due to the activation of a defence mechanism in the plants by the pre-applied Cd (“priming effect”). Confirmation of this effect requires further analysis. The results also suggest that glutathione plays a different role in Cd and pathogen variants. While in infected leaves, its antioxidant role is assumed; in plants exposed to Cd, it likely participates more in metal conjugation or sequestration through phytochelatins. Thus, in this study, we identified mechanisms that are more or less specifically involved in defence against Cd and powdery mildew. The changes in the parameters observed were dependent on the variety and the experimental variant. The interaction between these variables was also confirmed. The results obtained have the potential to predict the behaviour of oats not only under Cd or pathogen stress, but also under combined stress conditions. These findings may also contribute to increasing oat tolerance to adverse environmental factors, enhancing its phytoremediation potential, and reducing health risks associated with growing oats in Cd-contaminated soils.

## Supplementary Information

Below is the link to the electronic supplementary material.

Supplementary Material 1 (DOCX 13.2 KB)

Supplementary Material 2 (DOCX 16.8 KB)

Supplementary Material 3 (DOCX 2.02 MB)

## Data Availability

The data that support the findings of this study are available from the corresponding author, [Beáta Piršelová], upon reasonable request.
