# Supplementary material for: Study of selected mechanisms of oat tolerance to cadmium and powdery mildew
Source: Environ Sci Pollut Res Int. 2025 Oct 15;32(41):23540–56. doi: 10.1007/s11356-025-36951-x (PMC12553577; doi:10.1007/s11356-025-36951-x)
Supplement: Supplementary file 1 — Supplementary Material 1 (DOCX 13.2 KB) [file 11356_2025_36951_MOESM1_ESM.docx]

**Table S1** Summary of the results from the multifactorial ANOVA for physiological and biochemical parameters

| Parameter | Difference between variants (V) | | Difference between genotype (G) | Interaction (V * G) |
| --- | --- | --- | --- | --- |
| Cd content_Shoot | p < 0.001 | p < 0.05 | | p < 0.01 |
| Cd content_Root | p < 0.001 | p < 0.001 | | p < 0.001 |
| Ca content_Shoot | p < 0.001 | p < 0.001 | | n.s. |
| Lipid peroxidation_Leaves | p < 0.001 | p < 0.001 | | p < 0.001 |
| β-1,3-glucanase activity_ Leaves | p < 0.001 | p < 0.001 | | p < 0.001 |
| Catalase_ Leaves | p < 0.001 | p < 0.001 | | p < 0.001 |
| Polyphenols_Leaves | p < 0.001 | p < 0.001 | | p < 0.001 |
| Total glutathion_Leaves | p < 0.001 | p < 0.001 | | n.s. |
| Reduced glutathion (2GSH)_ Leaves | p < 0.001 | p < 0.001 | | p < 0.001 |
| Oxidized glutathion (GSSG)_ Leaves | p < 0.001 | p < 0.001 | | p < 0.001 |
| GSH/GSSG_Leaves | p < 0.001 | p < 0.01 | | p < 0.001 |
| Chlorophyll *a_*Leaves | p < 0.001 | p < 0.01 | | p < 0.001 |
| Chlorophyll *b*_Leaves | p < 0.001 | p < 0.001 | | p < 0.001 |
| Carotenoids_Leaves | p < 0.001 | p < 0.01 | | p < 0.001 |
| Total chlorophylls_Leaves | n.s. | p < 0.01 | | n.s. |

Cd (cadmium), Ca (calcium), p – significance
