# Supplementary material for: Study of selected mechanisms of oat tolerance to cadmium and powdery mildew
Source: Environ Sci Pollut Res Int. 2025 Oct 15;32(41):23540–56. doi: 10.1007/s11356-025-36951-x (PMC12553577; doi:10.1007/s11356-025-36951-x)
Supplement: Supplementary file 2 — Supplementary Material 2 (DOCX 16.8 KB) [file 11356_2025_36951_MOESM2_ESM.docx]

**Table S2** Summary of one-factor ANOVA results for individual physiological and biochemical parameters – comparison of differences between P, Cd, and P + Cd variants (Tukey's Test).

| Cultivar | | Aragon | | Bay Yan 2 | | Ivory | | Vaclav | | Racoon | |  | | Aragon | | Bay Yan 2 | | Ivory | | Vaclav | | Racoon | |  |
| --- | --- | --- | --- | --- | --- | --- | --- | --- | --- | --- | --- | --- | --- | --- | --- | --- | --- | --- | --- | --- | --- | --- | --- | --- |
| **Parameter** | **Variant** | | **Cd** | | **Cd** | | **Cd** | | **Cd** | | **Cd** | |  | | **P** | | **P** | | **P** | | **P** | | **P** | |
| Cd_Shoots | P+Cd | |  | |  | |  | |  | |  | |  | | n.d. | | n.d. | | n.d. | | n.d. | | n.d. | |
| Cd_Roots | P+Cd | |  | |  | | n.s | |  | |  | |  | | n.d. | | n.d. | | n.d. | | n.d. | | n.d. | |
| Chl*a_*Leaves | P | |  | | n.s. | |  | |  | |  | |  | | - | | - | | - | | - | | - | |
|  | P+Cd | |  | |  | |  | |  | |  | |  | | n.s. | | n.s. | | n.s. | | n.s. | | n.s. | |
| Chl*b_*Leaves | P | |  | | n.s. | |  | |  | |  | |  | | - | | - | | - | | - | | - | |
|  | P+Cd | |  | |  | |  | |  | |  | |  | | n.s. | |  | | n.s. | | n.s. | | n.s. | |
| Car*_*Leaves | P | |  | | n.s. n.s. n.s. | |  | |  | |  | |  | | - | | - | | - | | - | | - | |
|  | P+Cd | |  | | n.s. | |  | |  | |  | |  | | n.s. | | n.s. | | n.s. | | n.s. | | n.s. | |
| Ca*_*Leaves | P | | n.s. n.s. n.s. | | n.s. n.s. n.s. | | n.s. n.s. n.s. | | n.s. n.s. n.s. | | n.s. n.s. n.s. | |  | | - | | - | | - | | - | | - | |
|  | P+Cd | | n.s. n.s. n.s. | | n.s. n.s. | | n.s. n.s. | | n.s. n.s. n.s. | | n.s. n.s. n.s. | |  | | n.s. | | n.s. | |  | | n.s. | | n.s. | |
| MDA*_*Leaves | P | | n.s. n.s. n.s. | |  | |  | |  | |  | |  | | - | | - | | - | | - | | - | |
|  | P+Cd | | n.s. | | n.s. | |  | |  | |  | |  | | n.s. | |  | |  | |  | |  | |
| CAT*_*Leaves | P | |  | |  | | n.s. n.s. n.s. | | n.s. n.s. n.s. | | n.s. n.s. n.s. | |  | | - | | - | | - | | - | | - | |
|  | P+Cd | | n.s. | |  | | n.s. | | n.s. | | n.s. | |  | | n.s. | | n.s. | | n.s. | | n.s. | | n.s. | |
| GSH*_*Leaves | P | |  | | n.s. n.s. n.s. | |  | |  | |  | |  | | - | | - | | - | | - | | - | |
|  | P+Cd | | n.s. | | n.s. | | n.s. | | n.s. | |  | |  | |  | | n.s. | |  | |  | | n.s. | |
| GSSG*_*Leaves | P | | n.s. n.s. | | n.s. n.s. n.s. | | n.s. n.s. n.s. | |  | | n.s. n.s. n.s. | |  | | - | | - | | - | | - | | - | |
|  | P+Cd | | n.s. | | n.s. | | n.s. | | n.s. | |  | |  | |  | | n.s. | | n.s. | |  | | n.s. | |
| 2GSH*_*Leaves | P | | n.s. n.s. | | n.s. n.s. n.s. | | n.s. n.s. | | n.s. n.s. | | n.s. n.s. | |  | | - | | - | | - | | - | | - | |
|  | P+Cd | | n.s. | | n.s. | | n.s. | | n.s. | |  | |  | |  | | n.s. | |  | |  | | n.s. | |
| GSH/GSSG*_*Leaves | P | | n.s. n.s. n.s. | | - | | - | | n.s. n.s.n.s. n.s. | | n.s. n.s. n.s. | |  | | - | | - | | - | | - | | - | |
|  | P+Cd | | n.s. | | n.s. | | n.s. | | n.s. | |  | |  | | n.s. | | n.s. | | n.s. | | n.s. | |  | |
| POL*_*Leaves | P | |  | |  | |  | |  | |  | |  | | - | | - | | - | | - | | - | |
|  | P+Cd | |  | |  | |  | |  | |  | |  | | n.s. | |  | |  | | n.s. | |  | |
| GLU*_*Leaves | P | |  | |  | |  | |  | |  | |  | | - | | - | | - | | - | | - | |
|  | P+Cd | |  | |  | |  | |  | |  | |  | |  | |  | |  | |  | | n.s. | |

Significant at 󠇂 p < 0.05 (light gray), p < 0.01 (medium gray) and p < 0.001 (dark gray), n.s. – not significant
