# Supplementary material for: Study of selected mechanisms of oat tolerance to cadmium and powdery mildew
Source: Environ Sci Pollut Res Int. 2025 Oct 15;32(41):23540–56. doi: 10.1007/s11356-025-36951-x (PMC12553577; doi:10.1007/s11356-025-36951-x)
Supplement: Supplementary file 3 — Supplementary Material 3 (DOCX 2.02 MB) [file 11356_2025_36951_MOESM3_ESM.docx]

**Supplementary Figure 1**


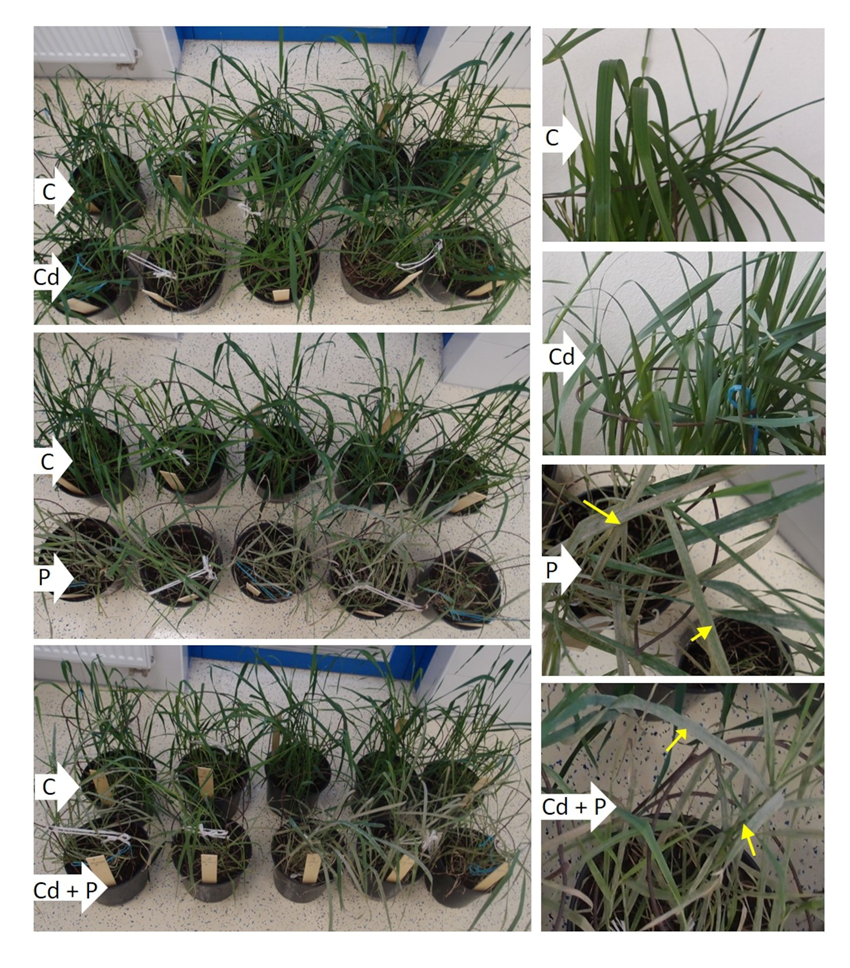


**Supplementary Figure 1:** Five oat varieties (Avena sativa) were exposed to cadmium (Cd), the pathogen Blumeria graminis (P), and their combination (P+Cd). The varieties, arranged from left to right, are: Aragon, Bay Yan 2, Ivory, Vaclav, and Racoon. On the right, close-up images of leaves from the control (C) and the Cd, P, and P+Cd treatments are shown. Yellow arrows indicate powdery mildew symptoms caused by Blumeria graminis (Photo: Katarína Bojnanská).
